# Interfractional Geometric Variations and Dosimetric Benefits of Stereotactic MRI Guided Online Adaptive Radiotherapy (SMART) of Prostate Bed after Radical Prostatectomy: Post-Hoc Analysis of a Phase II Trial

**DOI:** 10.3390/cancers13112802

**Published:** 2021-06-04

**Authors:** Minsong Cao, Yu Gao, Stephanie M. Yoon, Yingli Yang, Ke Sheng, Leslie K. Ballas, Vincent Basehart, Ankush Sachdeva, Carol Felix, Daniel A. Low, Michael L. Steinberg, Amar U. Kishan

**Affiliations:** 1 Department of Radiation Oncology, University of California, Los Angeles (UCLA), Los Angeles, CA 90095, USA; yugao@mednet.ucla.edu (Y.G.); SMYoon@mednet.ucla.edu (S.M.Y.); YYang@mednet.ucla.edu (Y.Y.); ksheng@mednet.ucla.edu (K.S.); VBasehart@mednet.ucla.edu (V.B.); ASachdeva@mednet.ucla.edu (A.S.); CFelix@mednet.ucla.edu (C.F.); dlow@mednet.ucla.edu (D.A.L.); MSteinberg@mednet.ucla.edu (M.L.S.); AUKishan@mednet.ucla.edu (A.U.K.); 2 Department of Radiation Oncology, University of Southern California, Los Angeles, CA 90089, USA; lballas@med.usc.edu

**Keywords:** stereotactic body radiotherapy SBRT, post-prostatectomy, prostate cancer, MR guided adaptive radiotherapy, MRgRT

## Abstract

**Simple Summary:**

Geometric variations of patients receiving stereotactic body radiotherapy (SBRT) after radical prostatectomy were evaluated in this study. With specific bladder filling and rectal emptying patient instructions, CTV shape and volume remain relatively stable and dose coverage is well maintained whilst maintaining OAR dose constraints in most treatment fractions. However, target coverage and OAR doses can be further improved by MR-guided online adaptive planning to account for interfractional geometric variations.

**Abstract:**

Purpose: To evaluate geometric variations of patients receiving stereotactic body radiotherapy (SBRT) after radical prostatectomy and the dosimetric benefits of stereotactic MRI guided adaptive radiotherapy (SMART) to compensate for these variations. Materials/Methods: The CTV and OAR were contoured on 55 MRI setup scans of 11 patients treated with an MR-LINAC and enrolled in a phase II trial of post-prostatectomy SBRT. All patients followed institutional bladder and rectum preparation protocols and received five fractions of 6−6.8 Gy to the prostate bed. Interfractional changes in volume were calculated and shape deformation was quantified by the Dice similar coefficient (DSC). Changes in CTV-V95%, bladder and rectum maximum dose, V32.5Gy and V27.5Gy were predicted by recalculating the initial plan on daily MRI. SMART was retrospectively simulated if the predicted dose exceeded pre-set criteria. Results: The CTV volume and shape remained stable with a median volumetric change of 3.0% (IQR −3.0% to 11.5%) and DSC of 0.83 (IQR 0.79 to 0.88). Relatively large volumetric changes in bladder (median −24.5%, IQR −34.6% to 14.5%) and rectum (median 5.4%, IQR − 9.7% to 20.7%) were observed while shape changes were moderate (median DSC of 0.79 and 0.73, respectively). The median CTV-V95% was 98.4% (IQR 94.9% to 99.6%) for the predicted doses. However, SMART would have been deemed beneficial for 78.2% of the 55 fractions based on target undercoverage (16.4%), exceeding OAR constraints (50.9%), or both (10.9%). Simulated SMART improved the dosimetry and met dosimetric criteria in all fractions. Moderate correlations were observed between the CTV-V95% and target DSC (R2 = 0.73) and bladder mean dose versus volumetric changes (R2 = 0.61). Conclusions: Interfractional dosimetric variations resulting from anatomic deformation are commonly encountered with post-prostatectomy RT and can be mitigated with SMART.

## 1. Introduction

While postoperative radiotherapy (RT) for adverse pathologic features and/or unfavorable genomic risk scores is the only curative intervention for patients with a rising PSA after radical prostatectomy, it is consistently underutilized at large [1]. The lack of utilization may be related to provider biases, challenges in the prolonged treatment course, and concerns about toxicity [2]. The logistical barriers to receipt of postoperative therapy might be mitigated by shorter RT courses. It has been suggested that prostate cancer (PCa) harbors a low alpha/beta ratio, suggesting that higher doses per fraction may allow isoeffective treatments over a compressed treatment schedule [3,4]. Ultrahypofractionated RT (≥5 Gy per fraction) has demonstrated non-inferior oncologic outcomes for intact PCa compared with conventional RT with an equivalent or favorable safety and toxicity profile [5,6,7]. As of 2020, stereotactic body radiotherapy (SBRT, or the delivery of ultrahypofractionated RT in five or fewer fractions) is now recognized as a standard-of-care option in the treatment of localized PCa [8].

In the context of postoperative RT, however, experience with hypofractionated RT of any form is still nascent, with long-term results of moderate hypofractionated RT just emerging [9,10,11] and randomized trials still underway. The major concerns with hypofractionated postoperative RT have been the “invisible” nature of the clinical target volume (CTV) which is significantly influenced by several deformable organs at risk (OARs), such as the bladder and rectum, and the possibility that the vesicourethral anastomosis may be more prone to toxicity than the native urethra [12,13,14,15]. Relevant to the first concern, a retrospective analysis of cone beam CT (CBCT) setup images of patients enrolled in a prospective phase II trial evaluating SBRT in the post-prostatectomy setting, found the interfractional change in CTV volume to be relatively small [16]. However, the CTV coverage goal was only met in 70% of fractions, suggesting the shape of the target and OARs may have a more direct dosimetric impact.

Online adaptive radiotherapy (ART) is one technology that might be particularly useful in the setting of a highly deformable target with deformable adjacent OARs [17]. Though commonly employed in post-prostatectomy RT, X-ray-based on-board imaging technologies, such as CBCT, are limited by its low soft-tissue contrast and image artifacts due to photon scatter, starvation, and motion degradation. Magnetic resonance image-guided radiotherapy (MRgRT) offers superior soft-tissue contrast compared with CBCT, making it a potential solution to better assess anatomical changes in post-operative prostate patients and provide better guidance for adaptive planning. Recent studies have shown that stereotactic MRI-guided online ART (SMART) can be safely and effectively delivered with dosimetric benefits for abdominal and pelvis tumors [18,19,20,21,22], but it has not been studied in post-prostatectomy RT. Here, we report volumetric and shape variations of the CTV and OARs of 11 patients who received MRI-guided post-prostatectomy SBRT in a single institution phase II trial, exploring the potential dosimetric benefits of SMART to compensate for these variations.

## 2. Materials and Methods

The institutional review board approved this retrospective analysis. The first 11 patients enrolled in a phase II dual-institution trial (NCT03541850) of post-prostatectomy SBRT who received treatment on a 0.35 T MR-guided linear accelerator (MR-LINAC, ViewRay MRIdian, ViewRay Inc. Cleveland, OH, USA) were included in this post-hoc analysis. Prior to salvage prostate bed SBRT, all patients underwent a technetium-99 bone scintigraphy scan, fluciclovine PET/CT scan, or prostate-specific membrane antigen (PSMA) PET/CT scan before MR and CT based simulation. For MR simulation, each patient was immobilized with a vacuum bag and a balanced steady-state free precession (bSSFP) sequence was acquired with 1.5-mm isotropic spatial resolution and a field of view of 50 × 45 × 43 cm^3^. The total acquisition time was 172 s. The prostate bed was contoured on the MR simulation scan to generate the clinical target volume (CTV) per RTOG consensus guidelines [23]. A 3 mm isotropic expansion was applied to the CTV to generate the planning target volume (PTV). The MR simulation scan was also used to contour OARs and was the primary image for treatment planning. A CT-based simulation was performed using the same immobilization device and images were deformed to the MR scan to provide electron density information for dose calculations.

Radiation treatment plans were designed to deliver 30–34 Gy in 5 fractions to the prostate bed such that 95% of the PTV received the prescription dose and all OAR dose constraints were met. If a gross tumor was visible on imaging, a gross tumor volume (GTV) was defined and expanded isotropically by 3–5 mm to form a PTVGross. This target could receive up to 40 Gy in five fractions with a simultaneous integrated boost. A treatment plan using 10–17 selected IMRT beams was generated using the ViewRay Treatment Planning System TPS (Version 5.4, ViewRay Inc. Cleveland, OH, USA) and the optimization was controlled by setting dose optimization parameters for the target and each OAR by the user. Dose calculation was performed with a grid resolution of 2 mm using a Monte Carlo-based algorithm that takes into account the MRI magnetic field effect on the dose distribution. Planning constraints for the OAR included bladder maximum dose (Dmax) < 35.7 Gy, V32.5 Gy < 35%; rectum Dmax < 35.7 Gy, V27.5 Gy < 45% and V32.5 Gy < 30%, and rectal wall V24 Gy < 50% that were derived from institutional constraints. For plans with the PTVGross, the bladder and rectum maximum dose were accepted if less than 39 Gy. The final prescription dose to the prostate bed was chosen in the range of 30–34 Gy to ensure all OAR dose constraints were met.

Prior to simulation and treatment on the MR-LINAC, all patients were instructed to follow a specific bladder and rectum preparation protocol. They were instructed to void their bladder one hour before each treatment and to drink 16–24 ounces of water to maintain a reproducible and comfortably full bladder. They were also instructed to take an enema the night before and on the morning of each treatment to ensure an empty rectum. After the initial setup, daily MR setup images were acquired using the same bSSFP sequence as the simulation. Soft tissue-based image registration was performed between the daily and planning MRI images focusing on the anterior rectal wall, and reviewed by a physician and physicist before proceeding with couch positioning correction and treatment initiation.

MR setup scans were obtained for these 11 patients and the prostate bed CTV and OAR were re-drawn on each MR scan by a single radiation oncologist (SY) and reviewed by the trial principal investigator (AUK). The percent changes in the interfractional CTV and OAR volumes were calculated, and the shape deformation of each structure was quantified by the Dice similarity coefficient (DSC) [24]. Online adaptive planning workflow was simulated in the ViewRay TPS by recalculating the initial treatment plan on the daily MR scan to generate a predicted plan, as described in detail elsewhere [25]. Dose metrics were extracted from the predicted plan to evaluate the delivered dose after image-guided couch correction. Pre-set dosimetric criteria were used to assess the necessity of adaptive planning. The criteria for adequate target coverage were CTV V95% ≥ 93% and PTV V95% ≥ 90%. The OAR adaptive constraints were the same as the planning constraints, except the Dmax for bladder and rectum were relaxed by 3% to 36.7 Gy considering the point dose uncertainty resulted from Monte Carlo dose calculation. If the predicted plan dose violated the preset dosimetric criteria, an adaptive plan was generated by re-optimizing the initial plan to the daily anatomy using the same beam parameters. Dosimetric parameters were extracted from the adaptive plan to evaluate the quality of adaption. Associations between dosimetric parameters and changes in volume and shape were assessed by regression analysis. Paired *t*-tests were performed to evaluate dosimetric changes between initial, predicted and adaptive plans.

## 3. Results

**Figure 1:**
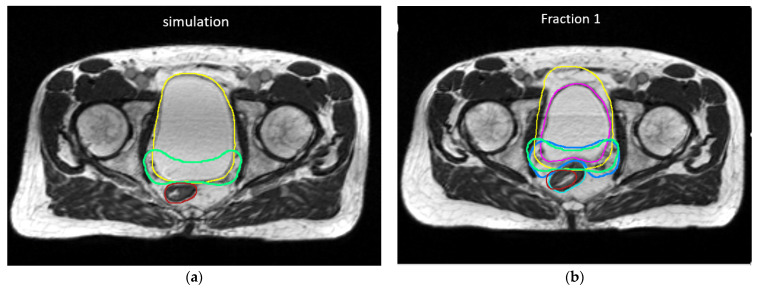
An example of CTV and OAR contours on (**a**) initial plan and (**b**) daily MRI on fraction 1 of a patient demonstrating relatively large interfractional shape variations in CTV and rectum and bladder. CTV: (Green–initial plan, blue–daily MR). Bladder: (yellow–initial plan, pink–daily MR); rectum (red–initial plan, cyan–daily MR).

A total of 55 MR setup scans of 11 patients were included for the analysis. Patient demographic and treatment characteristics can be found in Supplementary Table S1. The volumetric changes and shape variations as quantified by the DSC are summarized in Table 1. Overall, the interfractional CTV volume and shape remained stable with a median volumetric change of 3.0% (IQR −3.0% to 11.5%) and a median DSC of 0.83 (IQR 0.79 to 0.88). Relatively large volumetric changes were observed for the bladder (median −24.5%, IQR −34.6% to 14.5%) and rectum (median 5.4%, IQR −9.7% to 20.7%); however, shape changes of the bladder and rectum were moderate with a median DSC of 0.79 (IQR: 0.74 to 0.82) and 0.73 (IQR: 0.67 to 0.79), respectively. Figure 1 shows CTV and OAR contours of a patient exhibiting relatively large interfractional volume and shape changes.

Dosimetry from initial treatment plans, as well as the predicted and adaptive plans based on daily anatomy, are summarized in Table 2. After image-guided interfractional couch correction, the median change in the CTV V95% between predicted doses and the initial plan doses was −1.65% (*p* < 0.01). The target coverage goals of CTV V 95% ≥ 93% and PTV V95% ≥ 90% were met for 40 of 55 fractions (72.3%). Nine of 11 patients (81.8%) had an average CTV V95% ≥ 93% over 5 fractions. Despite relatively large volume changes, the median dosimetric changes in bladder and rectal doses were relatively small. Although there were no statistically significant differences in the mean bladder and rectal doses, relatively high maximum point doses were observed in the predicted plan for the bladder and rectum (*p* < 0.01). The average and median changes in the other dosimetric parameters were also minimal, except for the rectum and bladder V32.5 Gy, which showed a statistical difference (*p* < 0.01 and 0.02, respectively). Individual fraction analysis indicated that the bladder maximum dose constraints were met in 35 fractions (63.6%), and 43 fractions (78.2%) met the constraint bladder V32.5 Gy < 35%. The maximum rectum doses were found to exceed the constraint in 12.7% of the treatment fractions; however, the rectal volumetric dose constraints and the rectal wall V24 < 50% criterion were met in all the fractions. Overall, adaptive planning would have been deemed beneficial for 43 of 55 fractions (78.2%) based on target undercoverage (16.4%), exceeding OAR constraints (50.9%), or both the target and OAR criteria (10.9%).

**Figure 2:**
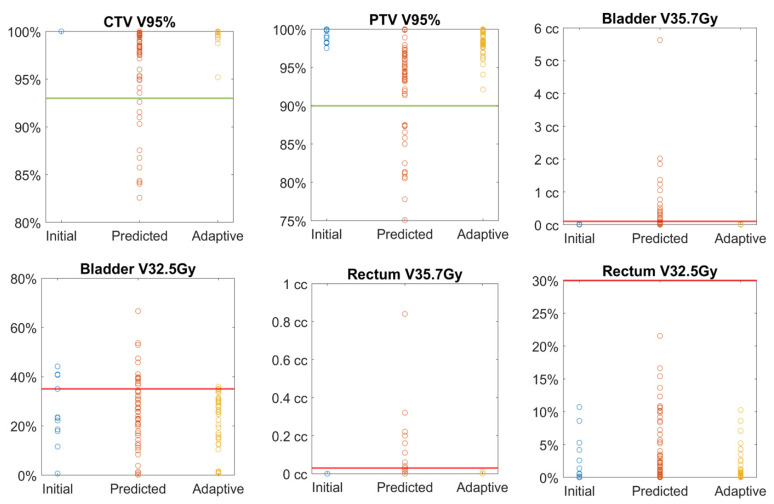
Comparison of target and OAR dosimetric metrics between initial, predicted and adaptive plan doses. The line in each plot represents the acceptable criterion. Bladder and rectum maximum doses are presented by V35.7 Gy < 0.03 cc.

Adaptive planning was performed for all 43 fractions violating the preset dosimetric criteria and led to the improvement of plan dosimetry as demonstrated in Figure 2. The CTV V95% ≥ 93% and PTV V95% ≥ 90% were met in all the adaptive plans and the median CTV V95% increased to 100% after adaptive planning, with no statistical difference when compared with the initial plan doses. Dosimetric constraints for OARs were met in all the adaptive plans except for two fractions in which the bladder V32.5 Gy was improved by the adaptive planning, but was still higher than the preset constraints. Constraints could be met after decreasing the fractional dose to 6.4 Gy from 6.6 Gy for these two fractions as per the original plan; such adjustments were allowed per protocol.

**Figure 3:**
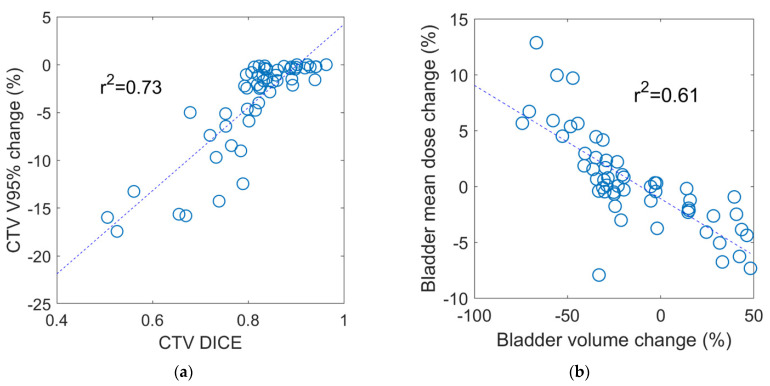
Correlation between dosimetric parameters versus interfractional geometric variations. (**a**) Change in CTV V95% versus CTV DICE (R2 = 0.73) (**b**) bladder mean dose change versus bladder volume change (R2 = 0.61). No strong correction was observed for the rest of the dosimetric metrics with neither volume nor shape changes.

Among the treatment fractions with acceptable target coverage, the median change in the CTV V95% was −1% (IQR −1.1% to 0.0) compared with −13.3% (IQR −15.8% to −9.2%) for those fractions with a CTV V95% < 93% (*p* < 0.001). Changes in CTV and OAR volumes were not significantly different between fractions with acceptable versus unacceptable CTV coverage. However, the CTV DSC was significantly smaller (median 0.72, IQR 0.58 to 0.76) in the fractions with unacceptable CTV coverage compared to a median of 0.84 (IQR 0.82 to 0.89) for those with acceptable coverage (*p* < 0.001), suggesting that CTV coverage is more related to changes in shape than volume. In addition, the rectum DSC was found to be significantly different between the two groups (*p* = 0.04) while there was no statistical difference in the bladder DSC. The rectum Dmax and bladder V32.5 Gy were the only dosimetric parameters that exhibited a significant difference between fractions with acceptable versus unacceptable CTV coverage, as shown in Table 3. Regression analysis identified a moderate correlation between the CTV V95% and CTV DSC (R2 = 0.73), as shown in Figure 3. A moderate correlation between the bladder mean dose and bladder volume change (R2 = 0.61) was also found, while no strong correlation was observed for the rest of the dosimetric metrics with neither volume nor shape changes.

## 4. Discussion

In this retrospective study, the volumetric and dosimetric variations of patients who prospectively received SBRT to the prostate bed on an MR-guided LINAC, with specific bladder and rectal filling instructions, were analyzed. Despite relatively large volumetric changes in the bladder and rectum, the CTV volumes and median dose remained stable. The vast majority (81.8%) of patients had acceptable target coverages averaged over all five treatment fractions, and among fractions that did not meet the coverage criteria, the median change in the CTV V95% was −13.3%. The CTV dosimetric changes were not directly correlated with volume changes of the bladder and rectum, but the shape of the rectum as represented by the DSC were statistically different between those fractions with acceptable and unacceptable CTV coverage, suggesting that target coverages may be more sensitive to changes in rectal shape. Our retrospective evaluation demonstrated that 78.2% of the treatment fractions could potentially benefit from adaptive planning. To our best knowledge, this is the first study demonstrating the dosimetric benefits of SMART to account for interfractional geometric variations in post-radical prostatectomy prostate bed radiotherapy.

The geometric and dosimetric variations without SMART are generally in line with a previously reported study based on CBCT analysis of a group of prostate bed patients enrolled in the same clinical trial, but treated with a conventional LINAC [16]. Both studies indicated that the CTV volumes and median dose remained stable despite relatively large interfractional volumetric variations in OARs. Approximately 72% of the patients in the previous CBCT analysis met the CTV V95% ≥ 93% constraints averaged over the treatment course, while a higher percentage (81.8%) of the patients were found to have acceptable coverage in this study. Slight improvements in OAR dosimetry were also observed in this study when compared with the prior study, with a higher percentage of patients meeting the bladder V32.5% < 35% (78% vs. 75%), rectum V32.5 Gy < 30% (100% vs. 98%), and rectum V27.5 Gy < 45% (100% vs. 99%). The favorable dosimetry, even without plan adaptation, might be attributed to the superior soft-tissue contrast of MR-guidance that is conducive to setup alignment based on the bladder–rectum interface, as opposed to the poor soft-tissue contrast of CBCT that necessitated bony anatomy-based registration. Neither study identified a strong correlation between CTV coverage with OAR volume changes. The current study proceeded further by also assessing shape variation with DSCs. The DSC ranged from 0.72−0.81 for OARs and CTV, suggesting relatively stable interfractional shape variations despite large volumetric changes. As mentioned earlier, the shape changes of the rectum were found to be slightly larger (median DSC = 0.68) for those fractions with unacceptable CTV coverage compared with those with acceptable CTV coverage (median DSC = 0.74, *p* = 0.04). The overall results emphasize the importance of implementing a stringent bladder and rectum preparation protocol when delivering SBRT. The target coverage and OAR sparing can be further improved by accounting for residual interfractional geometric variation through online SMART.

There is a paucity of data reporting the feasibility and efficacy of online ART for prostate cancers and most publications have focused on intact prostate treatment with SMART [26,27,28]. A prospective phase II study of SMART reported a low incidence of early toxicity in patients with localized PCa treated on the same 0.35T MR-LINAC as in this study, with an average treatment fraction time of 45 min [26]. Alongi et al. reported a single-institution prospective study using a 1.5T MR-LINAC with daily adaptive SBRT for 25 patients with localized PCa [27], with a median adaptive fraction time of 53 min. In another study of SBRT for localized PCa with daily adaptive planning on the same 0.35T MR-LINAC, the median treatment time was reported to be 45 min [28]. Of the treatment fractions, 76% were adapted, with 33.2% of them accounting for target coverage, 24.7% for OAR dose violations, and 36.3% for both. While adaptive planning workflow was almost identical for these two settings, it is difficult to directly infer and compare between clinical studies designed for intact prostate and post-operative settings.

There are several limitations to the present study. First, the number of patients is relatively small due to the natural accrual rate of patients on the current trial. This study also primarily focused on dosimetric evaluation through a simulated adaptive planning process. Despite these limitations, we were able to demonstrate the benefits of SMART for post-operative SBRT treatment even with a small cohort of patients. Based on the findings from this study, online adaptive planning has been actively performed for patients recently enrolled in the trial. Our initial experiences indicated that SMART for the post-operative setting was well tolerated by all patients, and we were able to perform in a similar fraction time as reported for the localized prostate treatment. The median time required for adaptive planning of the first five patients was found to be 17.2 min (range: 13–20 min) with a median beam delivery time of 14.8 min (range 13.3–18.2 min). Data is currently being collected for analysis of dosimetry, planning, and delivery aspects that will be presented in a later report. Although the interfractional geometric variations were found to be moderate in this study, the risk of intrafractional motion associated with increased treatment time should be considered. One of the advantages of MR-guided RT is that real-time MR cine imaging allows direct soft-tissue based gating of beam delivery. The rectum and bladder tissue interface detected on cine images was used as a gating surrogate in our treatment to account for potential intrafractional motion, which allows us to use a relatively small PTV margin of 3 mm in this study. To account for intrafractional motion without real-time motion tracking and gating, a larger PTV margin may be necessary or a faster delivery technique can be considered, e.g., VMAT delivery on a fast-rotating O-ring Linac system (Ethos). The dosimetric impact from intrafractional volume and shape change, and consequently the appropriate PTV margins are areas under investigation and will be reported in the future.

## 5. Conclusions

This retrospective geometric and dosimetric analysis of post-prostatectomy patients enrolled in a prospective trial receiving MR-guided SBRT confirms that when stringent bladder and rectum preparation protocols are in place, CTV volume and coverage remain stable without significantly overdosing OARs. Slight improvement in target coverage was observed, probably due to the better visualization of the patient anatomy with improved soft-tissue contrast offered by MR-guidance. Target coverage and OAR doses can be further improved by MR-guided online adaptive planning to account for interfractional geometric variations.

## Figures and Tables

**Table 1:** Interfractional volume changes and shape variations as quantified by Dice Similarity Coefficient (DSC).

Structure	Volume Change (%)				DSC			
	Mean	Median	Q1	Q3	Mean	Median	Q1	Q3
CTV	4.9	3.0	−3.0	11.5	0.81	0.83	0.79	0.88
Bladder	−14.8	−24.5	−34.6	14.5	0.76	0.79	0.74	0.82
Rectum	3.9	5.4	−9.7	20.7	0.72	0.73	0.67	0.79

CTV: clinical target volume; DSC: Dice Similarity Coefficient; Q1: 1st quartile; Q3: 3rd quartile.

**Table 2:** Dosimetric metrics of initial plan, predicted dose on daily anatomy and adaptive plan.

Dosimetric Parameter	Adaptive Criterion	Initial Plan				Predicted Plan				Adaptive Plan			
		Mean	Median	Q1	Q3	Mean	Median	Q1	Q3	Mean	Median	Q1	Q3
CTV mean dose (Gy)		34.3	34.3	33.8	34.7	33.8 *	33.8	33.3	34.6	34.2	34.2	33.5	34.9
CTV V95%	>93%	100.0	100.0	100.0	100.0	96.2 *	98.4	94.9	99.6	99.8	100.0	100.0	100.0
PTV mean dose (Gy)		34.0	34.0	33.4	34.6	33.5 *	33.5	32.9	34.4	34.0	33.9	33.4	34.7
PTV V95%	>90%	99.0	98.9	98.2	99.9	92.3 *	94.4	88.5	96.5	98.2 ^+^	98.4	97.8	99.5
Bladder mean dose (Gy)		20.7	22.6	15.2	25.0	21.1	21.9	17.7	24.9	22.6	23.3	19.9	25.5
Bladder Dmax (Gy)	<36.7 Gy	35.8	35.5	35.4	35.7	36.4 *	36.0	35.6	37.0	35.6 ^+^	35.4	35.1	35.7
Bladder V32.5Gy (%)	<35%	25.3	23.2	17.9	40.7	25.7	25.7	17.3	34.2	24.2	27.7	16.5	33.6
Rectum mean dose (Gy)		15.4	15.5	12.7	18.6	15.4	14.7	12.9	18.4	15.6	15.6	13.6	18.3
Rectum Dmax (Gy)	<36.7 Gy	34.5	35.0	33.4	35.2	35.5 *	35.2	34.5	36.2	34.1	34.2	33.5	34.7
Rectum V27.5Gy (%)	<45%	10.2	8.1	5.2	14.1	11.4	9.1	6.4	15.6	8.5 ^+^	7.2	4.4	11.0
Rectum V32.5Gy (%)	<30%	3.1	1.4	0.2	5.3	4.9 *	2.3	0.8	9.0	1.3 ^+^	0.4	0.1	1.1
Rectal wall mean dose (Gy)		15.6	15.3	12.7	18.7	15.3	14.6	12.7	18.3	15.7	15.8	13.5	18.2
Rectal wall V24Gy (%)	<50%	19.9	20.7	13.8	24.1	19.4	18.2	15.2	24.5	19.5	19.0	13.4	23.9

(* *p* <0.05 between initial and predicted plan doses, + *p* < 0.05 between initial and adaptive plan doses.)

**Table 3:** Interfraction volume and dosimetry among patients with acceptable and unacceptable CTV V95% coverage.

Dosimetric Parameter	Acceptable CTV95%					Unacceptable CTV95%					
	Mean	SD	Median	Q1	Q3	Mean	SD	Median	Q1	Q3	*p*-Value
CTV V95% (%)	98.36	1.75	98.91	97.71	99.75	87.33	3.51	86.75	84.24	90.82	<0.001
CTV Volume (cc)	113.66	26.60	122.77	91.70	131.89	114.28	25.02	123.80	89.78	129.85	0.945
Bladder volume (cc)	232.31	127.85	206.66	134.36	308.92	191.05	119.56	121.57	90.49	313.92	0.337
Rectum volume (cc)	67.22	14.29	63.94	58.37	74.22	66.33	13.52	64.08	59.21	71.81	0.853
CTV DSC	0.85	0.06	0.84	0.82	0.89	0.68	0.10	0.72	0.58	0.76	<0.001
Bladder DSC	0.77	0.11	0.79	0.75	0.82	0.74	0.07	0.76	0.70	0.79	0.490
Rectum DSC	0.73	0.08	0.74	0.69	0.80	0.68	0.08	0.68	0.62	0.73	0.042
Bladder mean dose (Gy)	21.50	5.22	22.60	17.94	25.33	19.70	4.65	20.38	16.20	22.68	0.300
Bladder Dmax (Gy)	36.25	0.94	36.02	35.65	36.59	37.24	2.82	37.41	34.53	39.76	0.052
Bladder V32.5 (%)	29.33	13.23	28.18	21.87	37.70	11.43	8.73	11.21	2.76	16.81	0.001
Rectum mean dose (Gy)	15.53	3.09	14.92	12.99	18.41	14.94	3.17	14.73	12.88	17.85	0.581
Rectum Dmax (Gy)	35.08	1.13	35.12	34.49	35.76	37.32	2.58	38.30	34.69	39.81	<0.001
Rectum V32.5 (%)	4.60	5.21	2.28	0.79	8.47	5.90	5.08	5.18	1.06	10.47	0.461
Rectm V27.5 (%)	11.05	7.33	8.55	6.34	14.81	12.61	5.47	13.63	7.22	16.62	0.512

## Data Availability

The data presented in this study are available in this article.

## Supplementary Materials

The following are available online at https://www.mdpi.com/article/10.3390/cancers13112802/s1, Table S1: Patient and treatment characteristics (n = 11).
