# Supplementary material for: Interfractional Geometric Variations and Dosimetric Benefits of Stereotactic MRI Guided Online Adaptive Radiotherapy (SMART) of Prostate Bed after Radical Prostatectomy: Post-Hoc Analysis of a Phase II Trial
Source: Cancers (Basel). 2021 Jun 4;13(11):2802. doi: 10.3390/cancers13112802 (PMC8200117; doi:10.3390/cancers13112802)
Supplement: Supplementary file 1 [file cancers-13-02802-s001.zip › cancers-1209447-supplementary.pdf]

Supplemental Table S1. Patient and treatment characteristics (n = 11)

Age	
Mean (SD)	66.5 (7.9)
Median (Range)	63.0 (63-73)
Risk Group, n (%)	
Favorable intermediate risk	1 (9.1)
Unfavorable intermediate risk	4 (36.4)
High risk	4 (36.4)
Very high risk	2 (18.2)
Gleason Grade Group	
Mean (SD)	3 (1.2)
Median (Range)	3 (2.5-4.5)
Pathologic T stage, n (%)	
2	4 (36.4)
3	7 (63.6)
Pre-operative PSA	
Mean (SD)	16.6 (21.0)
Median (Range)	10.1 (7.8-14.1)
Post-operative PSA	
Mean (SD)	0.3 (0.3)
Median (Range)	0.3 (0.2-0.4)
Radiation Intent, n (%)	
Salvage	11 (100.0)
Adjuvant	0 (0)
Dose to prostate bed	
Mean (SD)	32.2 (1.3)
Median (Range)	32.0 (32.0-33.0)
ADT use, n (%)	
Yes	5 (45.5)
No	6 (54.5)
